# A de novo variant in *OTX2* in a lamb with otocephaly

**DOI:** 10.1186/s13028-020-0503-z

**Published:** 2020-01-22

**Authors:** Julia Maria Paris, Anna Letko, Irene Monika Häfliger, Tanja Švara, Mitja Gombač, Primož Klinc, Andrej Škibin, Estera Pogorevc, Cord Drögemüller

**Affiliations:** 1 Institute of Genetics, Vetsuisse Faculty, University of Bern, Bremgartenstr. 109a, 3001 Bern, Switzerland; 2 Institute of Pathology, Forensic and Administrative Veterinary Medicine, Veterinary Faculty, University of Ljubljana, Gerbičeva 60, 1000 Ljubljana, Slovenia; 3 Clinic for Reproduction and Large Animals, Veterinary Faculty, University of Ljubljana, Cesta v Mestni log 47, 1000 Ljubljana, Slovenia; 4 Infrastructure Centre for Sustainable Recultivation Vremščica, Veterinary Faculty, University of Ljubljana, Gabrče 30, 6224 Senožeče, Slovenia; 5 Small Animal Clinic, Veterinary Faculty, University of Ljubljana, Cesta v Mestni log 47, 1000 Ljubljana, Slovenia

**Keywords:** Agnathia, Domestic animal, Microstomia, Precision medicine, Rare disease, Synotia, Whole-genome sequencing

## Abstract

**Background:**

Otocephaly is a rare lethal malformation of the first branchial arch. While the knowledge on the causes of otocephaly in animals is limited, different syndromic forms in man are associated with variants of the *PRRX1* and *OTX2* genes.

**Case presentation:**

A stillborn male lamb of the Istrian Pramenka sheep breed showed several congenital craniofacial anomalies including microstomia, agnathia, aglossia, and synotia. In addition, the lamb had a cleft palate, a small opening in the ventral neck region, a cystic oesophagus and two hepatic cysts. The brain was normally developed despite the deformed shape of the head. Taken together the findings led to a diagnosis of otocephaly. Whole-genome sequencing was performed from DNA of the affected lamb and both parents revealing a heterozygous single nucleotide variant in the *OTX2* gene (Chr7: 71478714G > A). The variant was absent in both parents and therefore due to a de novo mutation event. It was a nonsense variant, XM_015097088.2:c.265C > T; which leads to an early premature stop codon and is predicted to truncate more than 70% of the *OTX2* open reading frame (p.Arg89*).

**Conclusions:**

The genetic findings were consistent with the diagnosis of the otocephaly and provide strong evidence that the identified loss-of-function variant is pathogenic due to *OTX2* haploinsufficiency. The benefits of trio-based whole-genome sequencing as an emerging tool in veterinary pathology to confirm diagnosis are highlighted.

## Background

Otocephaly is a rare severe malformation of the first branchial arch [1, 2]. It is characterized by agnathia or mandibular hypoplasia, microstomia, aglossia or microglossia, and synotia and is often lethal [1–3]. Otocephaly is a key feature of two known rare disorders in man: agnathia-otocephaly complex (OMIM 202650) and microphthalmia with associated features including pituitary dysfunction (OMIM 610125). These mostly lethal phenotypes differ in severity and affected individuals rarely survive into childhood [4]. Two genes are known to be associated with these inherited disorders, the *orthodenticle homeobox 2* (*OTX2)* and the *paired related homeobox 1* (*PRRX1*) genes [1, 3, 4]. So far, all the *OTX2*-related cases were heterozygous and mostly due to dominantly acting de novo variants, whereas the pathogenic variants described in the *PRRX1* gene were either recessively or dominantly inherited [4]. Similar congenital anomalies including different syndromic forms of otocephaly occur also in domestic animals, for example in dogs (OMIA 001127-9615) [5], cattle (OMIA 001127-9913), and sheep (OMIA 000023-9940) [6–9]. In a single case of ovine agnathia karyotyping revealed a disease-causing reciprocal translocation [10]. So far, no single gene associated with otocephaly has been reported in any of these species.

## Case presentation

**Fig. 1:**
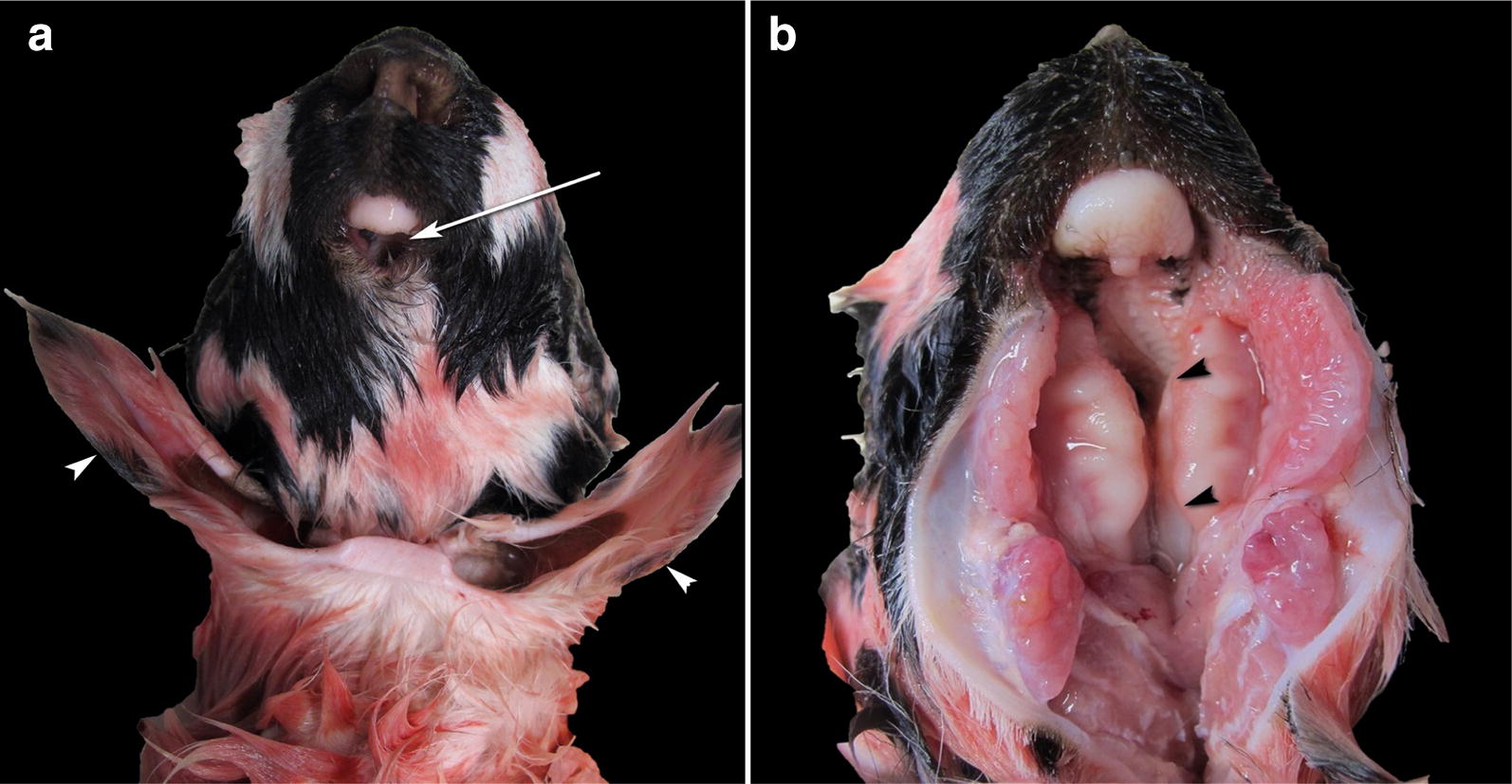
Otocephaly in an Istrian Pramenka lamb—gross pathology findings. **a** Ventral view of the head. The oral structures are very small (microstomia) (arrow) and the auricular pinnae (arrowheads) are located at the medioventral part of the head and neck (synotia). **b** Ventral view of the head with opened oral cavity. The palate is clefted (palatoschisis) (arrowheads) and the tongue is missing (aglossia). The lower jaw appears to be absent

**Fig. 2:**
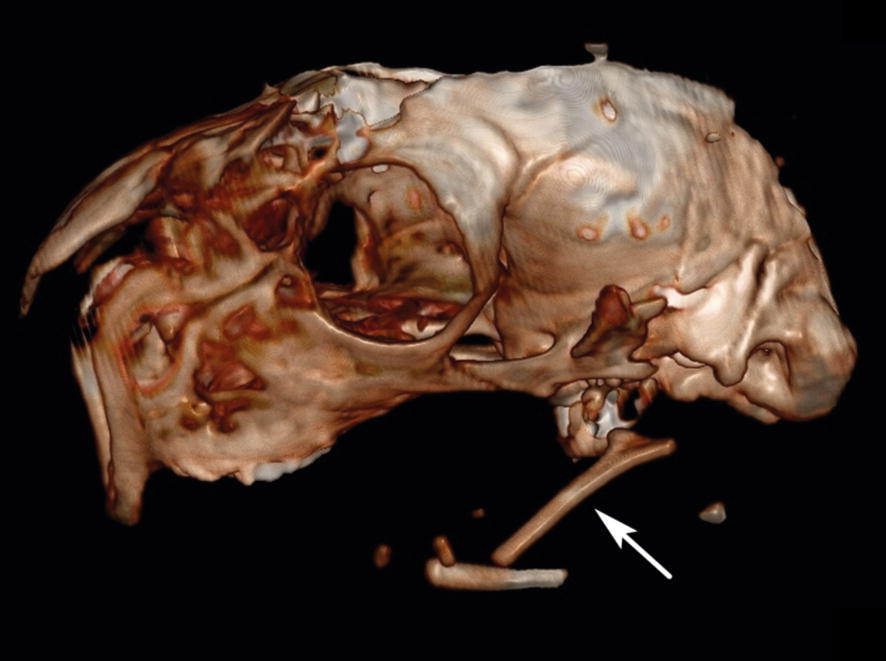
Otocephaly in an Istrian Pramenka lambs—CT findings. Lateral CT scan of the head shows deformed frontonasal bones and displacement of the hyoid bones (arrow). The following CT parameters were used: 160 mAs, 110 kV, 1.0 mm slice thickness, 1.0 mm reconstruction increment and beam pitch 1.3

In February 2018, an 8-year-old Istrian Pramenka ewe from a flock counting 500 sheep served by 16 different rams delivered a male lamb weighing 6 kg at term by caesarean section. The lamb died in less than a minute. At necropsy, severe craniofacial deformations were found. The lamb had dorso-ventrally deformed and shortened nasal region, very small oral aperture (microstomia), the tongue was missing (aglossia), and the hard palate was clefted (palatoschisis) (Fig. 1). The lower jaw was not found (agnathia). The auricular pinnae were located at the medioventral part of the head and neck (synotia) and without communication to the external auditory canal (*meatus acusticus externus*) (Fig. 1). Between the pinnae, there was an oval opening, measuring 1 × 0.5 cm, into a 2 cm long blind tubular structure. A later computed tomography (CT) scanning (Somatom Scope CT scanner, Siemens, Erlangen, Germany) of the head revealed deformed frontonasal bones and hyoid bones in abnormal position (Fig. 2). The oesophagus had a 1 cm long segmental stenosis and a proximal cystic dilatation. Additionally, two thin-walled cysts filled with yellow, translucent fluid were found on the surface of the liver. The brain and other organs were normally developed. The lungs had diffuse congenital atelectasis. Specimens of the brain, lung, heart, kidney, liver, adrenal gland, thymus and spleen were sampled for histopathology, fixed in 10% neutral buffered formalin, processed by routine methods, embedded in paraffin and sectioned at 4 µm and stained with haematoxylin and eosin. Histopathological examination revealed normally developed organs, complete pulmonary atelectasis, mild generalised congestion, focal haemorrhages in the meninges and thymus and mild hydropic degeneration of the hepatocytes. The gross lesions and CT imaging findings were consistent with otocephaly.

**Fig. 3:**
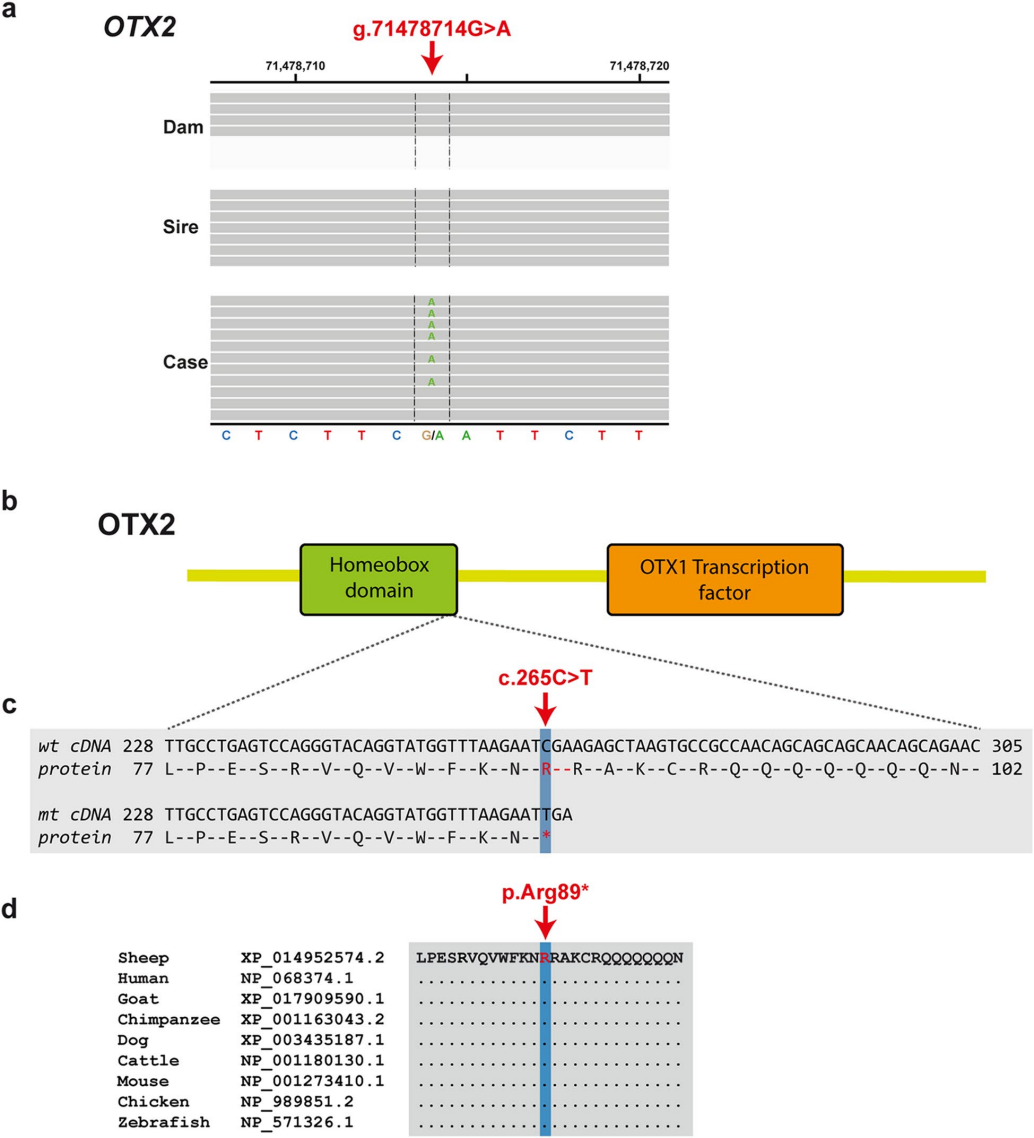
Otocephaly in an Istrian Pramenka lamb—genetic analyses. **a** IGV (12) screenshots of the affected lamb and its parents show the missense *de novo* variant in the *OTX2* present in the case and absent from both parents. **b** Schematic representation of the OTX2 protein showing the location of highly conserved domains. **c** cDNA and amino acid sequences in the region harbouring the described variant. **d** Conservation of the protein across multiple species

Genomic DNA of the lamb’s skin and from EDTA blood of all sampled adults including the dam and 16 possible fathers was isolated using standard protocols. All 18 animals were genotyped for single nucleotide variants (SNV) on Illumina OvineSNP50 BeadChip array. PLINK v1.9 [11] was used for quality control of the data and parentage confirmation. Pruning based on missing genotype calls per marker (> 10%) resulted in 41,571 markers in the final dataset. Paternity status was checked for all 16 potential sires of the affected lamb and resulted in the exclusion of 15 rams and validation of a single ram as the sire. Subsequently, whole-genome sequencing of the affected lamb and both parents was carried out. Therefore, Illumina TruSeq PCR-free libraries were prepared for sequencing 2 × 150 bp paired-end reads on an Illumina HiSeq 3000 instrument. Read mapping to the Oar_rambouillet_v1.0 ovine reference genome assembly and variant calling and annotation was done as described previously [12]. The genotypes of the affected lamb were compared with both parents and 54 additional sheep genomes of various breeds that had been sequenced in the course of other ongoing studies (European Nucleotide Archive accession no. PRJEB30931). Due to the strong effect of the mutation, it was hypothesized that a loss-of-function variant affecting the coding sequence of a gene most likely would be responsible for the disease. In addition, it was hypothesized that the mutant allele of the causative variant should be completely absent from all other 54 sheep genomes in the control sample set. Adopting an assumption of a possible recessive mode of inheritance, 12 missense variants were found to be homozygous in the affected offspring and heterozygous in the parents (Additional file 1). Genome-wide filtering for protein-changing sequence variants in the whole genome that were present heterozygous only in the affected lamb and homozygous wild type in the genomes of both parents, resulted in four variants representing putative de novo sequence variants (Additional file 1). The list of private protein-changing variants includes a de novo heterozygous nonsense variant in *OTX2* (Chr7: 71478714G > A), a gene which is associated with otocephaly in people. The Integrative Genomics Viewer (IGV) was used for visual inspection of the identified variants [13] and confirmed that the variant was absent in both parents without any evidence for low-level mosaicism in any of the parents (Fig. 3a). Therefore, this variant was finally considered as the candidate causal mutation.

The variant was predicted to produce an early premature stop codon truncating more than 70% of the *OTX2* open reading frame (p.Arg89*) and resulting in a significantly shorter protein lacking the C-terminal OTX1-transcription factor binding domain of the protein (Fig. 3b). In comparison to the normal 289 amino acid-long OTX2 protein, also the homeobox domain of the protein is truncated (Fig. 3c), which alters this evolutionary highly conserved and functionally important protein region (Fig. 3d). Therefore, the *OTX2*: XM_015097088.2:c.265C > T variant most likely leads to a complete loss-of-function.

## Discussion and conclusions

In the present study, a de novo nonsense variant in the ovine *OTX2* gene was identified in a newborn lamb with otocephaly. The syndromic phenotype was characterized by microstomia, aglossia, palatoschisis, agnathia and synotia, and differed from previous similar case reports in sheep with synotia, otocephalus aprosopus or acephaly [7–9]. In a single stillborn lamb with splanchnocranial anomalies that were classified as aprosencephaly and otocephaly, an association with the *OTX2* gene was ruled out [6]. Recently, morphological details of a single strophocephalic lamb with facial anomalies allocated to holoprosencephaly were presented but no molecular genetic examination was performed [14].

The reported *OTX2* variant introduces an early stop codon into the open reading frame. Although the expression of the mutant *OTX2* gene was experimentally analysed, it seems likely that the mutant transcripts will be subject to nonsense-mediated decay and that the *OTX2*:c.265C > T variant leads to a true null allele of the *OTX2* gene. Due to the lack of appropriate material the RNA could not be analysed experimentally to confirm this hypothesis. The variant was absent in both parents and therefore due to a de novo mutation event. This mutation might have occurred during meiosis of parental, most likely paternal, germ cells and subsequently passed to the offspring as a consequence of low-level mosaicism in one of the parents. No evidence for further cases in the flock was obtained and therefore, the mutation might also have occurred post-zygotically during early embryonic development of the affected lamb explaining this single case.

The *OTX2* homeobox gene plays a crucial role in craniofacial morphogenesis during early embryonic development. The *OTX2* gene encodes a transcription factor, which is essential for the early development of the embryonic head and later also for the development of the eye and brain [4]. The OTX2 protein belongs to the OTX group of signalling molecules with a DNA-binding homeodomain region, which are highly conserved across all species [4]. Mouse models confirmed the involvement of *OTX2* in pharyngeal arch formation and how its disruption leads to major craniofacial malformations [15].

On the basis of the combination of the available data, knowledge on *OTX2* function in other species, and the fact that the identified nonsense variant is likely to represent a loss-of-function mutation, the causality of the *OTX2*:c.265C > T variant for the observed otocephaly has been established beyond a reasonable doubt. This is in accordance with the described dominantly inherited loss-of-function variants in *OTX2* causing variable forms of agnathia-otocephaly complex, a pattern of malformations comprising mandibular hypoplasia/agenesis, ear anomalies, microstomia with oroglossal hypoplasia or aglossia, in humans (OMIM 600037) [3, 16], which were similar to the abnormalities observed in this lamb. Most of the *OTX2* variants identified in patients lead to a truncated or absent protein, and thus to OTX2 haploinsufficiency, e.g. as in the case of a heterozygous *OTX2* deletion associated with isolated mandibular dysostosis [17]. In a single human male fetus presented with agnathia, astomia and aglossia, absent pharyngeal floor, low posteriorly rotated, paramedian and convergent ears, a similar nonsense variant (p.Arg97*) at the corresponding position of the human *OTX2* transcript was described [3].

In summary, this study reports a heterozygous de novo variant in *OTX2* resulting in haploinsufficiency, in which there is no tolerance of loss-of-function variants in one copy of a gene that causes otocephaly. Recent large data from human genome sequencing studies presented in the Genome Aggregation Database (gnomAD) [18] showed that the pLI score, the probability of loss-of-function intolerance, for *OTX2* is > 0.9 meaning that *OTX2* falls into the class of loss-of-function haploinsufficient genes.

This study provides an example of a pathogenic disease causing variant underlying a sporadic syndrome observed in sheep. Germline genetic variation is a quite frequent causal factor in rare diseases also in domestic animals. Therefore, collecting of samples from parents in addition to the affected offspring to perform trio-based genetic analyses is a prerequisite for successful detection of the underlying genetic cause. Precision medicine by using whole-genome sequencing has been proven suitable in recent years to help diagnose rare diseases in human patients. Progress in the field of DNA sequencing is rapidly advancing and techniques can be successfully adapted for routine diagnosis in veterinary medicine, to both confirm genetic aetiology and to obtain an insight into the molecular mechanisms involved.

## Supplementary information

**Additional file 1.** List of private protein-changing sequence variants.

## Data Availability

The genomic data of the sequenced sheep genomes are available under study accession no. PRJEB30931 in the European Nucleotide Archive (https://www.ebi.ac.uk/ena; sample accessions of the studied parent–offspring trio: SAMEA5239895, SAMEA5239896 and SAMEA5239897). All references to the ovine *OTX2* gene correspond to the accessions NC_040258.1 (NCBI accession), XM_015097088.2 (mRNA), and XP_014952574.2 (protein). The reference genome assembly Oar_rambouillet_v1.0 and NCBI annotation release 103 was used for WGS.

## Abbreviations

CT: computed tomography
IGV: integrative genomics viewer
OMIA: online Mendelian inheritance in animals
OMIM: online Mendelian inheritance in man
OTX2: orthodenticle homeobox 2
PRRX1: paired related homeobox 1
SNV: single nucleotide variant
WGS: whole-genome sequencing
